# Potassium permanganate cleansing is an effective sanitary method for the reduction of bacterial bioload on raw *Coriandrum sativum*

**DOI:** 10.1186/s13104-018-3233-9

**Published:** 2018-02-13

**Authors:** Supram Hosuru Subramanya, Vasudha Pai, Indira Bairy, Niranjan Nayak, Shishir Gokhale, Brijesh Sathian

**Affiliations:** 1 Manipal College of Medical Sciences, Pokhara, Nepal; 2 Melaka Manipal Medical College, Manipal University, Udupi, India

**Keywords:** Coriander leaves, Cilantro, Contamination, Potassium permanganate

## Abstract

**Objective:**

Raw vegetables including flowers, leaves, stems, and roots are important carriers of food borne pathogens. We evaluated the bacteriological contamination of unwashed coriander leaves, and effectiveness of cleansing with 0.1% potassium permanganate solution as decontamination method.

**Results:**

Significant bacterial contamination including pathogens like *Salmonella* species and *Aeromonas* species were isolated from unwashed coriander leaves. Decontamination with 0.1% potassium permanganate was found to be more effective than three steps wash with sterile water.

## Introduction

Food-borne illness outbreaks are increasing globally, leafy green are well-recognized potential sources of bacterial food-borne infections [1]. Food safety has major implications for human health. Consumption of fresh, uncooked or partially cooked vegetables plays important role in the outbreaks of food borne infections [2, 3]. Safety in consumption of leafy green vegetables is a growing concern, particularly in developing countries. These are likely to get contaminated during cultivation, transport and storage [2–4]. A recent report of the 2007 FAO/WHO meeting provided an overview of the pathogens most commonly associated with fresh fruit and vegetables (http://www.who.int/foodsafety). Fresh vegetables and herbs were implicated as vehicles for the transmission of food borne microbial infections worldwide [3]. An outbreak of Shiga toxin producing *Escherichia coli* infections attributed to consumption of leafy vegetables caused nearly 200 laboratory confirmed illnesses, 100 hospitalizations and few deaths in the USA in 2006 [5]. While leafy vegetables are an important part of a healthy diet, consumption of uncooked contaminated flavouring agents is a major concern.

Disinfection is important to sanitize the green leafy vegetables and fruits. Many researchers have found the efficacy of various disinfectants to reduce the bioload on ready to eat leafy green. It has been known that potassium permanganate (KMnO_4_) solution is one such effective disinfectant, and many researchers used it against a wide range of microorganisms. Soriano et al. [6] and Amoah et al. [7] used potassium permanganate solution in disinfecting lettuce, and found a significant decrease in the bacterial load on these green leaves. Like lettuce, Coriander or cilantro (*Coriandrum sativum*) is a very commonly used edible leaf which is often garnished on cooked dishes. Besides, it is favored as a delicacy in most Asian dishes due to its unique flavor. Thus we planned to study the microbial quality of coriander leaves as these leaves in the raw state could be contaminated with one or more pathogenic microorganisms. Washing with plain water before use as is followed in many household cooking practices, cannot assure freedom from pathogens. Thus there seems to be a need for decontamination of coriander leaves before consumption. This study was, conducted to determine the microbiological quality of raw cilantro leaves and to assess the effectiveness of washing with 0.1% KMnO_4_ solution as decontamination procedure.

## Main text

### Materials and methods

A total of 35 bundles of coriander leaves were purchased from 35 vendors stationed at 17 different vegetable markets located all around the Udupi town, India. Samples were collected in twenty visits from the vendors over a period of one and a half months between February and March 2016. Samples were collected in pre-sterilized plastic bags and transported with minimum delay to the microbiology laboratory. The roots of the specimen were trimmed aseptically and the leaves and parts of stem were chopped with sterile scissors. Chopped leaves were then aliquoted into 3 portions A, B and C each weighing approximately 25–30 g. These were processed separately.

Portion A of chopped leaves was inoculated into Selenite F broth (approximately 20–25% by volume) and incubated at 37 °C for 6 h for enrichment of fecal pathogens like *Salmonella* species and *Shigella* species [8]. Loopful of Selenite F broth was sub-cultured on xylose lysine deoxycholate agar and examined after 14–18 h of incubation at 37 °C. Colonies suggestive of *Salmonella* species or *Shigella* species were further studied and characterized morphologically and biochemically by VITEK automated system [8].

Portion B of samples were analysed quantitatively for total count by adding 50 ml of sterile distilled water and manual shaking for 3 min, with approximately 20 shakes per min. The wash content (water) was subjected to a serial tenfold dilution (1:10–1:10,000 in sterile peptone water) and 0.01 ml volume of the neat and each dilution were inoculated onto MacConkey agar plates for determining the CFU/ml of the sample. The wash process was repeated three times and the content of water was inoculated in a similar manner. All the plates were inoculated at 37 °C for 18–24 h. The total bacteriological count was noted during each wash process. The different morphotypes of bacteria were further identified using standard biochemical reactions [8].

Portion C was dipped in 0.1% potassium permanganate solution (KMnO_4_) with manual shaking of approximately 20 shakes per min, allowing a total of 10 min contact time at room temperature. The solution was decanted and fresh sterile distilled water was added to the leaves and decanted to remove the traces of KMnO_4_. The refilled fresh water was shaken vigorously and used to determine the colony count by adopting the same procedure as mentioned above for portion B. All experiments were conducted in duplicates and at room temperature of approximately 23 °C.

#### Data analysis

Data obtained were analyzed by the IBM SPSS Statistics 20 software from IBM Corporation, Armonk, New York, USA. 95% confidence interval used to generalize all the mean counts. Viable counts were normalized by log transformation before applying t test to compare with base line. p value < 0.05 considered as statistically significant.

## Results

**Fig. 1:**
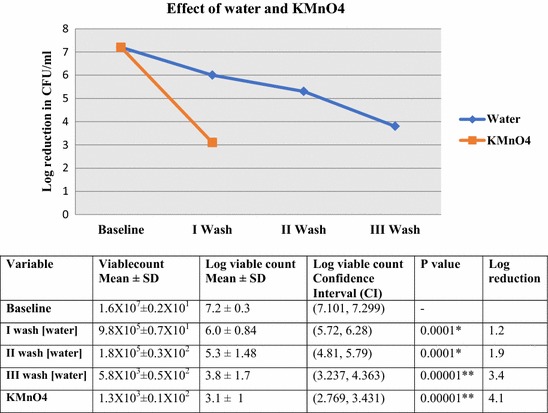
Viable bacterial counts of coriander leaves after sterile water and KMnO_4_ wash

*Salmonella typhi* was isolated from one sample of portion A (1/35). In portion B all 35 samples showed bacterial contamination with the mean viable count of 1.6 × 10^7^ CFU/ml (7.2 log10 CFU/ml). Coliform gram negative bacilli were found in all the samples. The contingency in Table 1 shows the number and percentage isolation of various bacteria. Potential pathogen *Aeromonas* spp. were detected in 6(17%) samples. Two or more than two types of bacteria were isolated from 95% (33/35) of the samples. Washing steps of portion B with water revealed a mean 1.2 log reduction in the viable bacterial load during first wash followed by 1.9 log reductions after second wash and 3.4 log reductions after the third wash. Compared with the findings of Portion B, the viable count in portion C (KMnO_4_ wash) was reduced by 4.1 log. The log reduction of bio-load with each wash steps using water and single decontamination step with potassium permanganate is depicted vide Fig. 1.

**Table 1:** Bacteria isolated from coriander leaves of different samples (Portion B)

Sample no.	Bacteria	Number of bacterial isolates (%) N = 35
1	*Salmonella* spp.	1 (2.85)
2	*Klebsiella* spp.	22 (62.85)
3	*Enterobacter* spp.	21 (60)
4	*Escherichia coli*	12 (34.28)
5	*Citrobacter* spp.	8 (22.85)
6	*Providencia* spp.	2 (5.71)
7	*Morganella* spp.	1 (2.87)
8	*Serratia* spp.	3 (8.57)
9	*Pseudomonas* spp.	4 (11.42)
10	*Aeromonas* spp.	6 (17.14)
11	Non fermenters	8 (22.85)
12	*Staphylococcus* spp.	4 (11.42)

### Discussion

Consumption of raw fruits and vegetables have been implicated in several food borne illnesses [9, 10]. In developing countries, untreated waste water irrigation and manure as fertilizers contribute to contamination of vegetables and herbs by human and animal fecal flora [11]. If the cold chain is not maintained during transportation and storage, the bacterial multiplication may occur. Several studies demonstrated the presence of potential pathogens on fresh vegetables and leaves [3, 7, 11]. In this study, we have demonstrated the contamination of cilantro with various fecal floras which may colonize or cause infection in humans. Detection of enteric pathogens like *Salmonella* spp., *Aeromonas* spp. and *Escherichia coli* is a major concern. The leaves could have been contaminated in the farms during cultivation probably due to waste water irrigation. Mritunjay and Kumar [12] reported contamination of vegetable salads with pathogens like *Salmonella* and Enterohaemorrhagic *Escherichia coli.* They were of the view that the enteric bacteria would multiply in absence of effective decontamination measures during the harvesting, processing and packing. The diarrheagenic potential of *E coli* obtained in our case could not be determined due to paucity of resources. The *E coli* Q157:H7 could survive in raw vegetables at ambient temperature retaining its infectivity. Serious illnesses ranging from bloody diarrhea to hemolytic uremic syndrome can occur due to consumption of contaminated raw vegetable [13].

In this study *Pseudomonas aeruginosa, Providenia* spp.*, Morganella* spp. and *Serretia* spp. were detected among 11.42, 5.71, 2.87 and 8.57% of the specimen respectively. Michael et al. [14] also reported these organisms in raw vegetables. *Pseudomonas aeruginosa* being a common natural inhabitant of soil is likely to contaminate vegetables. *Morganella* and *Providencia* being part of normal gastrointestinal tract flora of man and animals could contaminate the soil because of open field defecation. These opportunistic pathogens can cause septicemia in immune compromised individuals. Therefore attention should be focused on the food processing, storage and transport in order to eliminate potential pathogens.

Washing the leafy vegetables with water, supposed to flush out the microorganisms is considered a satisfactory household decontamination method. But this method is influenced by various factors like availability of sufficient clean water and the original bioload on the food items. Washing with water several times is shown to reduce the bioload significantly [7], only when the washing steps are multiple, stringent and vigorous. In the present study, as well as in a similar study conducted earlier [6], sterile distilled water was used for the initial step of washing, which may not appear to be an ideal technique as this could damage the bacterial cells. However, such initial exposure to sterile distilled water for a very limited time period might not have affected the quality of the result.

Many researchers worked on the efficacy of various sanitizers and decontaminants like NaCl solutions, vinegar, combined salt and vinegar, laundry detergent, household bleach products, iodine, trisodium phosphate etc. in various concentrations and with various contact time, but none of them could completely eliminate fecal coliform populations from vegetables and fruits [9].

Of the available methods of decontamination of leafy greens, chlorination was reported to be effective, but it affects the structural integrity of leaves. The potency of common bleach is variable and unreliable. Low dose gamma irradiation was another method that efficiently reduced the microbial load while retaining the quality of leafy vegetables eaten raw [15].

Potassium permanganate solution was known to reduce the counts of pathogenic bacteria and parasites on fresh vegetables and fruits [7, 16, 17]. In this study we have observed that viable bacterial load on cilantro was reduced in a significant number by KMnO_4_ washing.

KMnO_4_ in low concentrations is often used as antiseptic mouths wash for maintenance of oral hygiene and also as a purifying agent of well water used for drinking purpose. According to recent WHO guidelines, KMnO_4_ in a concentration as low as 1:10,000 could be safe for topical application on open wounds [18, 19], the lethal effect being far apart corresponding to a dose as high as 10 gm [20]. Thus washing edible items with very low concentration (0.1%) KMnO_4_ solution followed by plain water wash may not have any adverse effect on human health.

The role of education, training and awareness among the producers, handlers and consumers is important in order to improve product safety. Washing vegetables before consumption is an important approach for health risk reduction. This study showed that coriander leaves have high bacterial contamination, inclusive of pathogens like *Salmonella* spp.*, Aeromonas* spp. and *E coli*. The decontamination effect of potassium permanganate solution on the leaves was proved to be better than the three wash steps with plain water. The effect of the KMnO_4_ solution on human health and taste or aroma of the leaves has not been studied in this work; this emphasized the need for further research in certain areas to facilitate the effective sanitary method.

### Conclusion

The current work highlights the bacterial diversity and load on raw coriander leaves. The potassium permanganate solution at 0.1% concentration for minimum 10 min contact time proved to be an efficient and easy method to significantly reduce the bacterial load. This can be used as an alternative to or in combination with plain water washing. To the best of our knowledge, this is the first ever study demonstrating the efficacy of potassium permanganate solution as a cleanser for cilantro.

## Limitation

This study involves only in vitro cultivable bacteria. Bacterial isolates were not further characterized for virulence properties or drug resistance. Effectiveness of KMnO_4_ washing was tested for single concentration and contact time.

## Abbreviations

KMnO_4_: potassium permanganate
FAO: Food and Agriculture Organization of the United Nations
WHO: World Health Organization
CFU: colony forming units
NaCl: sodium chloride
